# Comparison of stool collection on site versus at home in a population-based study: Feasibility and participants’ preference in Pretest 2 of the German National Cohort

**DOI:** 10.1007/s00103-014-2051-z

**Published:** 2014-10-08

**Authors:** A. Schultze, M.K. Akmatov, M. Andrzejak, N. Karras, Y. Kemmling, A. Maulhardt, S. Wieghold, W. Ahrens, K. Günther, H. Schlenz, G. Krause, F. Pessler

**Affiliations:** 1 Department of Epidemiology, Helmholtz Centre for Infection Research, Inhoffenstraße 7, 38124 Braunschweig, Germany; 2 Leibniz Institute for Prevention Research and Epidemiology—BIPS, Bremen, Germany; 3 TWINCORE Center for Experimental and Clinical Infection Research, Hannover, Germany; 4 University of Applied Sciences and Arts, Hannover, Germany; 5 Hanover Medical School, Hannover, Germany

**Keywords:** Feasibility study, Population-based cohort, Native stool, Acceptance, Collection site, Machbarkeitsstudie, Bevölkerungsbasierte Kohorte, Nativstuhl, Akzeptanz, Ort der Stuhlgewinnung

## Abstract

**Background:**

For certain laboratory investigations it is necessary to obtain native stool samples and process them within a narrow time window at the point of contact or a nearby laboratory. However, it is not known whether it is feasible to obtain stool samples from asymptomatic individuals during an appointment in a study center (SC). We therefore compared participants’ preference, feasibility and acceptance of stool sample collection during the appointment at the study center (on-site sampling) to collection at home after the appointment.

**Methods:**

The study was conducted at two sites in Northern Germany (Bremen, *n* = 156; Hannover, *n* = 147) during the Pretest 2 phase of the German National Cohort (GNC), drawing upon a randomly selected population supplemented by a small convenience sample. In the study center, the participants were given the choice to provide a stool sample during the appointment or to collect a sample later at home and return it by mail.

**Results:**

In all, 303 of the 351 participants (86 %) of Pretest 2 at these sites participated in this feasibility study. Only 7.9 % (24/303) of the participants chose on-site collection, whereas 92 % (279/303) chose at-home collection. There were significant differences between the two study sites in that 14 % (21/147) of participants in Hannover and 2 % (3/156) of participants in Bremen chose on-site collection. Compliance was high in both groups, as 100 % (24/24) and 98 % (272/279) of participants in the on-site and at-home groups, respectively, provided complete samples. Both methods were highly accepted, as 92 % of the participants in each group (22/24 and 227/248) stated that stool collection at the respective site was acceptable.

**Conclusion:**

When given a choice, most participants in this population-based study preferred home collection of stool samples to collection in the study center. Thus, native stool samples for immediate processing in the study center may potentially be obtained only from a subpopulation of participants, which may lead to selection bias. Home collection, on the other hand, proved to be a highly feasible method for studies that do not require freshly collected native stool.

## Abstract (de)

**Hintergrund:**

Für die Durchführung bestimmter Laboranalysen wird Nativstuhl benötigt, der zeitnah vor Ort oder in einem nahegelegenen Labor verarbeitet werden muss. Bislang ist nicht bekannt, ob es möglich ist, Stuhlproben von gesunden Probanden während ihres Aufenthaltes im Studienzentrum zu erhalten. Wir haben daher die Präferenz von Studienteilnehmern sowie die Machbarkeit und Akzeptanz der Stuhlprobengewinnung während des Termins im Studienzentrum im Vergleich zur Stuhlprobengewinnung zu Hause nach dem Termin im Studienzentrum untersucht.

**Methoden:**

Die Studie wurde während des Pretest 2 der Nationalen Kohorte in 2 Studienzentren Norddeutschlands (Bremen, n = 156; Hannover, n = 147), basierend auf einer Zufallsstichprobe und ergänzt durch ein kleines Convenience Sample, durchgeführt. Im Studienzentrum wurden die Probanden vor die Wahl gestellt, entweder die Stuhlproben während des Termins im Studienzentrum abzugeben oder später zu Hause und die Stuhlproben per Post zurückzusenden.

**Ergebnisse:**

Für eine Teilnahme an der Machbarkeitsstudie haben sich 303 von 351 (86 %) der Pretest-2-Probanden beider Studienzentren entschieden. Nur 7,9 % (24/303) der Probanden entschieden sich für die Gewinnung der Stuhlprobe im Studienzentrum, während 92 % (279/303) die Heimgewinnung vorzogen. Es gab signifikante Unterschiede zwischen den beteiligten Studienzentren insofern, als 14 % (21/147) der Probanden in Hannover und 2 % (3/156) der Probanden in Bremen die Stuhlgewinnung im Studienzentrum gewählt haben. Die Compliance war in beiden Gruppen hoch: 100 % (24/24) der Probanden mit Stuhlsammlung im Studienzentrum und 98 % (272/279) der Probanden mit Stuhlsammlung zu Hause haben vollständige Stuhlproben abgegeben. Es gab eine hohe Akzeptanz für beide Methoden: Jeweils 92 % der Probanden beider Gruppen (22/24 und 227/248) bewerteten die Gewinnung im Studienzentrum bzw. zu Hause als akzeptabel.

**Diskussion:**

Vor die Wahl gestellt, haben sich die meisten Probanden in dieser bevölkerungsbasierten Studie für die Gewinnung von Stuhlproben zu Hause statt im Studienzentrum entschieden. Daher können Nativstuhlproben wohl nur von einer kleinen Gruppe von Probanden für die sofortige Verarbeitung im Studienzentrum gewonnen werden, was möglicherweise zu Selektionsverzerrungen führt. Andererseits hat sich die Heimgewinnung als sehr zuverlässige Methode für Fragestellungen, die keine frisch gewonnenen Nativstuhlproben erfordern, erwiesen.

Collection of biosamples has become an increasingly frequent feature of prospective population-based studies worldwide, including the German National Cohort (GNC). Stool samples could potentially be used for studies regarding early detection of gastrointestinal neoplasms, nutritional and metabolic parameters, and gastrointestinal microflora and pathogens. However, thus far only a few prospective population-based cohort studies have been conducted in which stool samples were requested, for instance with the aim to estimate the incidence and species distribution of gastrointestinal pathogens in the community [1–3]. These studies involved collection of stool samples at home during symptoms of a gastrointestinal infection, with reported participation proportions varying between 9 and 42 % . For some laboratory investigations, such as detection of certain metabolites or cultivation of certain microorganisms, it is necessary to obtain native stool samples and process them within a narrow time window at the point of contact or a nearby laboratory. Home collection would therefore not be appropriate in such scenarios. However, it is not known whether it is feasible to obtain stool samples from healthy individuals during a prespecified narrow time window such as a participant’s appointment in a study center. Indeed, even though on-site stool collection is common place in medical settings, its feasibility in population-based studies comprised of individuals without evidence of gastrointestinal infection has not been tested. Using a population-based study design embedded within the Pretest 2 phase of the GNC, we therefore compared feasibility and participants’ preference of on-site and at home stool collection in asymptomatic individuals. We found that the majority of participants preferred home collection but that both methods met high compliance and acceptance by the individuals who selected the respective method (see also article by A. Kühn et al. in this issue).

## Methods

### Stool collection at baseline in Pretest 2

**Fig. 1:**
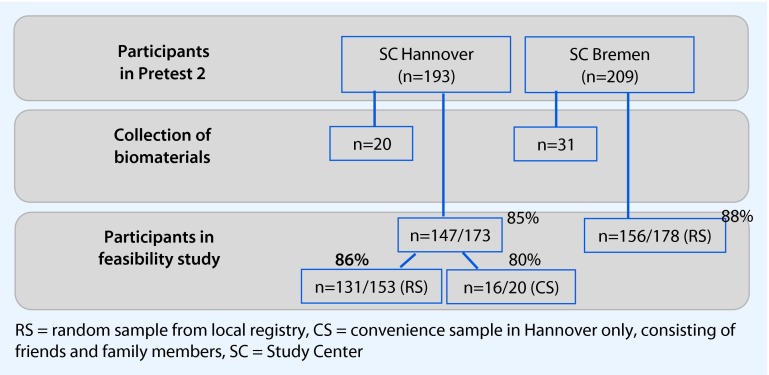
Flow chart of the recruitment for the feasibility study.

A schematic of the recruitment for Pretest 2 and for the present feasibility study is shown in Fig. 1. About 200 participants were to be recruited for the pilot studies of Pretest 2 by each of the 18 study centers across the nation. The collection of all biomaterials that were to be included in the planned baseline assessments of the GNC (blood, urine, feces, saliva, nasal swabs) was to be tested in a small subpopulation of 20 participants per study site. Among these “n = 20” study participants, stool collection followed the currently planned standard operating procedures (SOPs) for main recruitment for the GNC, i.e. the participants were asked to collect a stool sample at home in the evening or morning before the appointment and then bring it to the study center. The stool collection kit (including one tube for native stool, one tube containing DNA stabilizer, and a short acceptance questionnaire) was sent to this subgroup by mail prior to the appointment. These participants were excluded from the present feasibility study.

### Recruitment for the feasibility study

In two study centers (Bremen, Hannover) the remaining participants (*n* = 178 in Bremen, *n* = 173 in Hannover) were informed upon arrival in the study center that they could choose between (1) collecting the stool samples in the study center during the appointment (anticipated to last about 3.5 h; “on-site collection”) and (2) collecting the samples at home and mailing them directly to the Helmholtz Centre for Infection Research (“home collection”). Stool collection kits and standard operating procedures (SOPs) for pre-analytical processing were essentially the same as in the planned baseline assessments of the GNC. In communicating the project to the participants it was explained that the collection of fresh stool samples on site would be of great scientific benefit because fresh material offers the best quality for certain laboratory analyses. Additionally, in Hannover the convenience of on-site collection was emphasized in that all materials were ready to be used, and no additional time and effort would be required later at home.

### Procedure for on-site collection

The procedure for on-site collection was explained along with a short pictorial description. The restrooms for the participants were provided with the stool collection kit including two collection tubes (one for native, one for DNA-stabilized stool) and illustrated instructions for collecting the stool samples. A storage surface or container for deposition of samples was provided in the restrooms. Upon leaving the restroom, the participant notified the study nurse, who then carried the specimen to the laboratory for pre-analytical processing. The participants were asked to complete a brief acceptance questionnaire. The laboratory staff completed a biosample protocol after pre-analytic processing.

### Procedure for home collection

If a participant chose home collection, this procedure was explained using a pictorial description. The participants were given a stool collection kit including two collection tubes (one for native, one for DNA-stabilized stool), an illustrated description of how to collect the stool samples, a short questionnaire, and a prepaid shipping box to return the samples to the Helmholtz Centre for Infection Research.

### Acceptance questionnaires

The questionnaire for on-site collection comprised seven questions, the questionnaire for home collection six questions. Both questionnaires contained questions about the acceptability of on-site stool collection versus collection at home or, if applicable, the reasons for not participating or failing to participate. The date and time of sample collection had to be indicated. Furthermore, the questionnaire for home collection included a question about the storage condition of the specimens before mailing them.

### Materials and sample collection procedure

**Fig. 2:**
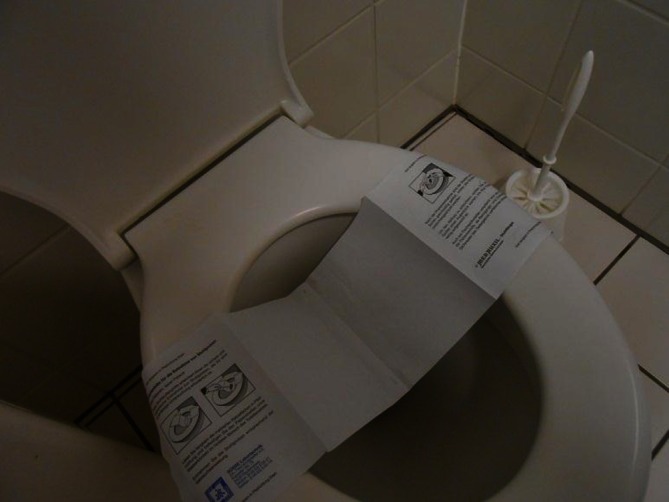
Stool collector used for on-site and home collection made of water-soluble recycling paper (Med Auxil)]

The stool kit included a stool collector (Süsse Stuhlfänger MED AUXIL 150 × 470), two stool collection tubes with integrated spoons, disposable gloves, and a plastic specimen bag. One stool collection tube (S1) was meant for the collection of native stool (Sarstedt Stuhlsammelgefäß 80.734.001 76/20 PP steril), the other tube (S2) was prefilled with stool DNA-stabilizer for collection of DNA-stabilized stool specimen (Stratec molecular stool collection tube, #1038111200). The directions for safe and hygienic fecal collection were printed on the stool collector in words as well as in pictograms. Participants were instructed to carefully unfold the adhesive surface of the stool collector in the direction of the arrows and to attach it to the posterior surface of the toilet seat (Fig. 2). The two collection tubes were to be put within reach and the disposable gloves donned. The spoon of the first collection tube (S1) was to be used to collect small (pea-sized) stool samples from three different spots of the specimen and transfer them into S1. The instructions for sampling and filling tube S2 were basically the same. After filling tube S2, the stool material was to be mixed carefully with the liquid inside (DNA-stabilizer), the lid tightly closed and the collection tube vigorously shaken about ten times. The collection tubes were then to be placed into the specimen bag to prevent liquid spilling. The stool collector and the remaining fecal matter were to be flushed away after sample collection was completed.

### Ethics approval

Participants gave written informed consent. The study was approved by the Ethics Committee of the State Board of Physicians of the German Federal State of Lower Saxony (Ethikkommission der Ärztekammer Niedersachsen).

## Results

### Participation proportions

The overall participation proportion in Pretest 2 in both study centers was just under 20 %. Whereas all participants in Bremen were recruited out of a random sample drawn from the local residents’ registry, 14 % of the participants in Hannover originated from a convenience sample of friends and colleagues. As the study protocol for the main phase of the GNC allows for random samples only, all results will be discussed mainly in this respect. In total, 351 of 402 Pretest 2 participants in both study centers were invited to participate in this “On-site stool collection” study. Considering the random sample only, the overall participation proportion in this study was 87 % (287/331), with nearly identical participation proportions per center of 86 % in Hannover and 88 % in Bremen.

Table 1 contains the data on age and sex of the participants in this feasibility study compared to all Pretest 2 participants. Females were somewhat overrepresented in the stool study regardless of sampling method or study center. The overall correlation between sex and participation was significant for the random sample in that women were more likely to participate than men (*p* = 0.001, x^2^ test).

**Table 1:** Gender and age distribution—participants of this feasibility study versus Pretest 2

	SC Hannover			SC Bremen		Total	
	RS	incl. CS	Pretest 2^a^	RS	Pretest 2^a^	RS	Pretest 2^a^
Female (%)	54.2	57.1	50.0	57.7	53.6	56.1	52.0
Male (%)	45.8	42.9	50.0	42.3	46.4	43.9	48.0
20–29 years (%)	5.3	6.1	7.2	3.8	3.8	4.5	5.3
30–39 years (%)	8.4	10.2	9.6	6.4	5.3	7.3	7.2
40–49 years (%)	26.0	25.9	28.3	25.0	25.8	25.4	26.9
50–59 years (%)	26.0	25.9	25.9	28.8	32.5	27.5	29.6
60–69 years	34.0	32.0	28.9	35.9	32.5	35.2	30.9
Age median of participants	53	53	52	54	53	54	52
Age median of non-participants	47	46	–	52		50	

*RS:* random sample, *CS:* convenience sample, *SC:* study center

^a^Random sample only

The age distribution of the participants reflects, overall, the targeted age distribution of the GNC, which is weighted toward the age group 40 to 69 years. In Hannover the age group 20–29 years was slightly underrepresented in this feasibility study compared to the Pretest 2 population; conversely, the age group 60–69 years was somewhat overrepresented in both study centers. Participation proportions were highest (94 %, 101/107) in the 60- to 69-year-old group and lowest (81 %, 73/90) in the age group 40–49 years.

### Preferred site of stool collection

In both centers combined, 7.9 % (24/303) of the participants preferred on-site collection and 92 % (279/303) home collection. There was a considerable difference between the two sites in that only 1.9 % (3/156) of the participants in Bremen but 14 % (21/147) of the participants in Hannover chose on-site collection. There were no significant socio-demographic differences between participants who chose on-site and those who chose at home collection (data not shown).

### Compliance with on-site and home stool collection

In all, 98 % (296/303) of the participants provided complete stool samples (S1 and S2) with 100 % complete samples in the on-site collection group and 98 % (272/279) in the home collection group. In the latter group, 1.4 % (4/279) of the participants provided only one tube (either S1 or S2) and three participants failed to return any stool sample because of problems with the stool collector. Upon receipt of the home-collected samples in the laboratory, the following irregularities were observed: eight samples lacked an ID, four samples had not been placed in the specimen bags provided with the kit, and three samples were mixed with urine.

### Time from collection to receipt in the laboratory—on-site sampling

**Fig. 3:**
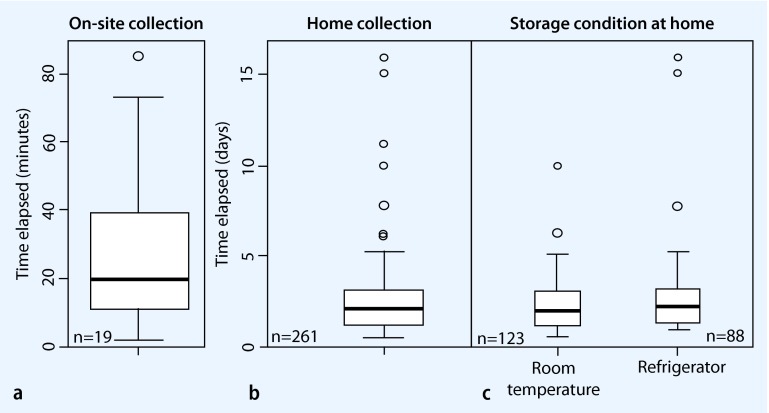
Variation in time elapsed between sample collection and initiation of frozen storage. **a** *On-site collection*. The data were calculated using the time of collection recorded by the participants and the time of placement in the laboratory freezer recorded by the laboratory staff. **b** *Home collection*. The data were calculated using the time of collection recorded by the participants and the time of receipt of the shipped package in the laboratory of the Helmholtz Center for Infection Research. **c** *Storage condition at home*. The data were calculated using the time of collection recorded by the participants and the time of receipt of the shipped package in the laboratory of the Helmholtz Center for Infection Research (base: all responses given in regard of storage condition of sample at home before shipping, *n* = 211/261).

Data about the time span from sampling to freezing the stool material were available for 91 % (19/21) of stool samples collected at the study center in Hannover. The median time was 20 minutes (range 2–85 min). Of these samples 63 % (12/19) were frozen within 2–30 min, and 37 % (7/19) within 30–85 min. (Fig. 3).

### Home collection

The date of sample collection was reported by 95 % (265/279) of individuals who collected the stool sample at home. The first 3  days of the week were the preferred collection time in the following order: Tuesdays (21 %), Wednesdays (19 %) and Mondays (16 %). Instruction concerning a preferred day for sending samples had not been given. There were no significant differences between the two study centers in this regard. Most participants (*n* = 238) indicated the storage condition of the samples before sending them to the Helmholtz-Centre for Infection Research. More than half of the samples (53 %) were stored at room temperature, 37 % in the refrigerator and 9.7 % were mailed immediately. Overall, 94 % (261/279) of the participants kept records of date and time of sampling, and the time elapsed between specimen collection to returning the samples to the laboratory could therefore be calculated (Fig. 3). The median transit time was 49.5 h (range 12 h–16 days). There were no significant differences between the study centers. Considering the time elapsed stratified by storage condition prior to shipping, significant differences were observed between samples stored at room temperature and samples stored in the refrigerator. Finally, 52 % (64/123) of samples which were stored at room temperature were returned within 48 h versus 40 % (35/88) of samples stored in the refrigerator (x^2^ = 12.44, df = 4 and *p* = 0.01).

### Acceptance questionnaire

The short acceptance questionnaire was answered by 90 % (272/303) of all participants, 96 % (23/24) of the on-site collection group, and 89 % (248/279) of the home collection group. The data per study center are shown in Table 2. Stool collection was accepted equally well in both groups, with 92 % of the on-site collection group and 92 % of the home collection group stating that stool collection at the respective site was acceptable. Of the participants who collected stool samples at home, 82 % (203/248) assessed sampling at home to be more acceptable than at the study center. But 15 % (37/248) of all participants (14 % in Hannover and 16 % in Bremen) agreed or agreed partly with the statement that stool collection at the study center might be more acceptable than at home. Of those participants who collected stool samples at the study center, 46 % (11/24) found this approach more acceptable than home collection, 29 % (7/24) agreed partly that stool collection at home might be more acceptable than stool collection at home. In all, 7.7 % (19/248) of participants in the home collection group reported major problems with the stool specimen collector, which failed in its purpose, ruptured, or clogged the toilet. No such comments were made by participants in the on-site collection group.

**Table 2:** Acceptance of stool collection

	SC Hannover	SC Bremen	Total
Stool collection at home			
Written information was comprehensible (yes)	94 %	95 %	95 %
… was acceptable (yes)	92 %	91 %	92 %
… was easy (yes)	85 %	82 %	83 %
… is an intrusion into my privacy (yes/partly)	9.6 %	15 %	13 %
… is more acceptable than at the study center (yes)	80 %	83 %	82 %
Stool collection at the study center is more acceptable than at home (yes/partly)	14 %	16 %	15 %
Comments of participants:			
Stool specimen collector dissolved, tore, plugged the toilet	9.6 %	6.3 %	7.7 %
**N**	**104**	**144**	**248**
Stool collection at the study center			
Verbally given information was comprehensible (yes)	91 %	100 %	92 %
Written information was comprehensible (yes)	86 %	100 %	88 %
… was acceptable (yes)	91 %	100 %	92 %
… was easy (yes)	86 %	100 %	88 %
… is an intrusion into my privacy (yes/partly)	14 %	–	13 %
… is more acceptable than later at home (yes)	48 %	33 %	46 %
Stool collection at home is more acceptable than at the study center (yes/partly)	29 %	33 %	29 %
N	21	3	24

*SC:* study center

## Discussion

In this population-based study on participants’ choice of the site for collecting a stool sample, we found that the great majority preferred home collection to on-site collection, although satisfaction, acceptance and compliance were equally high in both groups.

### Differences in participants’ choices of stool collection site

This study clearly demonstrates that it is possible to obtain a stool sample from the great majority of randomly selected participants who do not have a personal benefit from donating the sample (such as receiving a medical diagnosis or a medical intervention for gastrointestinal complaints). However, the overwhelming preference for home collection indicates that it may be possible to obtain fresh native stool samples from only a small subpopulation of participants, which may result in selection bias. What might be some of the reasons for the strong preference for home collection? Home collection clearly offers a greater degree of privacy and control over the sampling procedure. Moreover, many participants may not have anticipated being able to have a bowel movement during the approx. 3.5 h in the study center. Given the small size of the on-site collection group it was not possible to identify any significant differences between the two groups that might predict a selection bias in future studies. But individuals with less frequent bowel movements or even constipation would likely have chosen home collection, whereas those with softer stools and more frequent bowel movements might have chosen on-site collection. Thus, systematic biases relating to factors affecting stool composition and bowel habits might be introduced into both groups. Clearly this question would need to be addressed in better powered studies before on-site collection of stool could be included in the GNC or similar cohort studies.

### Differences between the two study sites

There were great differences between the two study sites in that a much higher percentage of participants chose on-site collection in Hannover than in Bremen. A likely explanation for this difference is that the study personnel in Hannover readily provided information about potential advantages of on-site collection to the participants. In Bremen, on the other hand, communication regarding the study was limited to the predefined text explaining that fresh stool material offers the best quality for scientific purposes. No further encouragement for on-site sampling was given in Bremen.

### Time between stool sample collection and completion of pre-analytical processing

In both groups, there was great variation in the time elapsed between completed collection and placement of the specimens in the laboratory freezer. Surprisingly, even in the on-site collection group several samples were frozen after more than 30 min. It is not possible to assess the impact, if any, of these apparent delays on the quality of the samples, as this would clearly depend on the planned investigations. But these findings do indicate that time from collection to freezing should be documented accurately and that staff training and optimizing workflow in the study center should be used to minimize and standardize the time required for pre-analytical processes. The differences in shipping time of the home-collected specimens were substantial, too, but are not likely to influence results of DNA-based analyses, as it is known that DNA is stable for several days in the preservative used [4, 5].

### Limitations of the study

This study is clearly limited by two factors. First of all, the sample size was not sufficient to identify significant differences between the individuals opting for on-site vs. home collecting. Secondly, we could not study the true collection proportion of native stool samples in the study center, as participants also had the choice of home collection. A study not giving the option of home collection would be needed to assess the success of on-site sampling more accurately.

## Conclusion

This study clearly demonstrated the feasibility of collecting stool samples in a population-based study and also identified participants’ homes as the preferred site for stool collection. Future studies should be directed at identifying differences between individuals who prefer on-site collection from those who prefer at home collection, in order to identify any selection bias, which will likely exist.

*Lessons drawn for the main recruitment phase of the GNC*

The results of this feasibility study solidified the previously made plans to use home collection as the main approach to collecting stool samples.

## References

[1] Wheeler JG, Sethi D, Cowden JM (1999). Study of infectious intestinal disease in England: rates in the community, presenting to general practice, and reported to national surveillance. The infectious intestinal disease study executive. BMJ.

[2] de Wit MA, Koopmans MP, Kortbeek LM (2001). Sensor, a population-based cohort study on gastroenteritis in the Netherlands: incidence and etiology. Am J Epidemiol.

[3] Tam CC, Rodrigues LC, Viviani L (2012). Longitudinal study of infectious intestinal disease in the UK (IID2 study): incidence in the community and presenting to general practice. BMJ Open Access.

[4] Nechvatal JM, Ram JL, Basson MD (2008). Fecal collection, ambient preservation, and DNA extraction for PCR amplification of bacterial and human markers from human feces. J Microbiol Methods.

[5] Carozzi FM, Sani C (2013). Fecal collection and stabilization methods for improved fecal DNA test for colorectal cancer in a screening setting. J Cancer Res.

